# Comparison Between Robotic and Conventional Laparoscopic Hysterectomy Performed Using Single‐Port Approach

**DOI:** 10.1111/ases.70044

**Published:** 2025-03-16

**Authors:** Akiko Ohwaki, Kyohei Takada, Arata Kobayashi, Mayuko Ito, Ryoko Ichikawa, Hironori Miyamura, Haruki Nishizawa

**Affiliations:** ^1^ Department of Obstetrics and Gynecology Fujita Health University School of Medicine Aichi Japan

**Keywords:** da Vinci SP surgical system, gynecological uterine disease, hysterectomy, minimally invasive surgery, single‐port surgery

## Abstract

**Introduction:**

Robotic surgery is being rapidly implemented globally, and new robotic surgery techniques are being developed. The da Vinci SP surgical system, a new robotic surgery system using a single‐port approach, was introduced for the first time in Japan, and its surgical results were compared with those of the conventional single‐port plus one‐port laparoscopic hysterectomy.

**Methods:**

The study included 20 patients who underwent single‐port robotic hysterectomy using the da Vinci SP surgical system (SP‐RH) between March 2023 and December 2023, and 37 patients who underwent single‐port plus one‐port laparoscopic hysterectomy (SP + 1‐LH) between March 2018 and December 2023. The surgical outcomes and complications were retrospectively compared.

**Result:**

When the SP‐RH group was compared with the SP + 1‐LH group, intraoperative blood loss was observed to be significantly lower in the SP‐RH group. However, no difference in the incidence of intraoperative complications between the two groups was observed. Furthermore, when comparing postoperative inflammatory responses, C‐reactive protein levels were significantly lower in the SP + 1‐LH group on the third day after surgery, but no other differences were observed.

**Conclusion:**

This study demonstrated that single‐port robotic hysterectomy using the da Vinci SP surgical system can be safely introduced and performed in clinical settings. The da Vinci SP surgical system, which uses a single‐port platform, can be used in minimally invasive surgeries as a novel operational system.

## 1 Introduction

Robotic surgery is being rapidly implemented globally, and new robotic surgery systems are being introduced continuously. Our hospital introduced the da Vinci SP surgical system (da Vinci SP), a new model from Intuitive Surgical Inc., in March 2023. The da Vinci SP is a surgical platform that enables surgery to be performed by installing a dedicated port with a single abdominal wall incision and by inserting a camera and three instruments from the same site. It is gaining attention as a minimally invasive robotic surgery system that differs from previous robotic surgery techniques using four robotic arms. Single‐port robotic surgery is advantageous because it is performed through a single incision. It produces more cosmetically pleasing results and can be performed in narrow spaces, such as in cases of oral surgeries [1, 2].

In conventional laparoscopic surgery, the concept of reduced port surgery intending to reduce incisions was also introduced from multiport laparoscopic surgery. Since 2010, dedicated equipment for single‐port laparoscopic surgery has been developed and clinically implemented. Single‐port laparoscopic surgery has gradually expanded its application from ovarian disease to uterine disease in the field of gynecology. However, it did not lead to the alleviation of the pain, and no validation results were reported showing a clear superiority of the procedure over multiport laparoscopic surgery in surgical outcomes [3, 4, 5]. Since then, the frequency of single‐port laparoscopic surgery has gradually decreased due to the complexity of the procedure, such as the interference of forceps and cameras, difficulty in securing a surgical space, and difficulty in acquiring surgeons who require extensive training [6, 7, 8, 9].

Our institution also introduced single‐port laparoscopic surgery around 2010. However, considering it unsuitable for learning surgical techniques and continuing education, we modified the procedure and transitioned to single‐port plus one‐port laparoscopic surgery (single‐port laparoscopic surgery with one additional auxiliary port) around 2015. This single‐port plus one‐port laparoscopic surgery is a method that optimizes the cosmetic results and the ease of use of forceps, and it has gained recognition as a surgical technique with a single‐port approach [10, 11, 12, 13, 14].

While laparoscopic surgery evolved toward minimally invasive procedures, robotic surgery was introduced, and comparative studies between laparoscopic and robotic surgery became popular. Previous comparisons of laparoscopic and robotic surgery in gynecologic diseases have often reported comparable surgical outcomes and complication rates [15, 16]. However, all of the analyses on robotic surgeries were for multiport cases, and no robotic surgery system with a single‐port platform had been developed. The da Vinci SP is a robotic system with a unique single‐arm design that eliminates interference between forceps, which has been a drawback of single‐port laparoscopic surgery and realizes precise and stable operations unique to robotic surgery. The da Vinci SP is a new robotic surgical system using a single‐port approach. As no reports have compared single‐port plus one‐port laparoscopic surgery with single‐port robotic hysterectomy using the da Vinci SP, the evaluation of invasiveness is essential. Therefore, this study aimed to compare the short‐term clinical outcomes of these two techniques performed at our institution.

## 2 Materials and Methods

**FIGURE 1:**
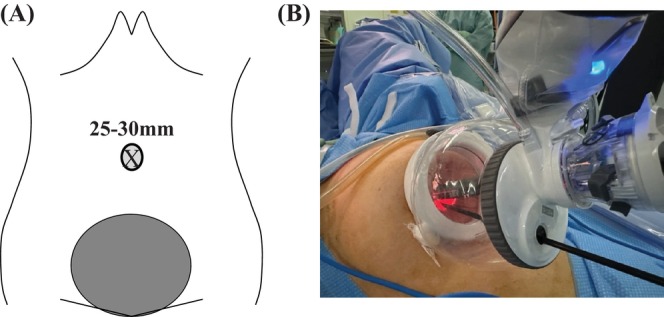
For the port of the SP‐RH group, a 25‐mm transverse incision was made in the umbilical region, and an SP Access Port Kit was placed at the same site. (A) Abdominal wall incision and (B) port placement in single‐port robotic hysterectomy using the da Vinci SP.

**FIGURE 2:**
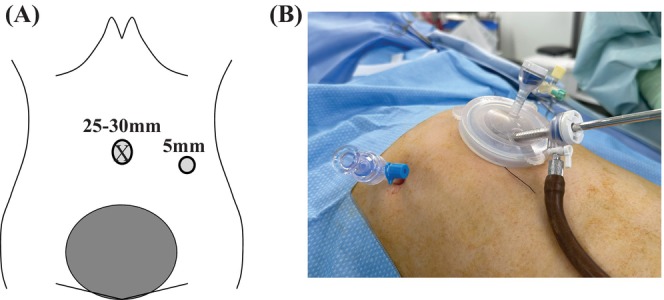
For the port of the SP + 1‐LH group, a 25‐ to 30‐mm transverse incision was made in the umbilical region to place an EZ access kit, and two 5‐mm trocars were inserted at the same site in addition to one 5‐mm trocar inserted in the left lower abdomen as an auxiliary port. (A) Abdominal wall incision and (B) port placement in single‐port plus one‐port laparoscopic hysterectomy.

The study included 20 cases of single‐port robotic hysterectomies performed using the da Vinci SP (SP‐RH) technique from March 2023 to December 2023, and 37 cases of single‐port plus one‐port laparoscopic hysterectomies (SP + 1‐LH) performed from March 2018 to December 2023 at our hospital for benign gynecological and uterine diseases such as uterine fibroids and adenomyosis. Prior to the surgery, the differences between conventional multiport surgery and single‐port surgery, as well as the advantages and disadvantages of laparoscopic surgery and robotic surgery, were explained to eligible patients, and the patient selected the surgical method according to their preference. For port placement in the SP‐RH group, a 25‐mm transverse incision was made in the umbilical region, and the SP Access Port Kit (Intuitive Surgical Inc., Sunnyvale, USA) was placed in the incision site (Figure 1). For port placement in the SP + 1‐LH group, a 25‐ to 30‐mm transverse incision was made in the umbilical region, an EZ access kit (Hakko Co. Ltd., Nagoya, Japan) was placed in the incision site, two 5‐mm trocars were inserted at the same site, and one 5‐mm trocar was inserted as an auxiliary port in the left lower abdomen (Figure 2). The energy devices used for the tissue incisions were SP Monopolar Curved Scissors or SP Fenestrated Bipolar for the SP‐RH group, and HARMONIC ACE (ETHICON, New Jersey, USA) was used for the SP + 1‐LH group. Surgical procedures and operations after placing the Access Port Kit were performed in the same way. The removal of the uterus was performed vaginally in both cases, with a two‐layer suture at the vaginal stump.

Following data were extracted from medical records and compared between the two groups: specimen weight, total operation time, port placement time, uterus out time, estimated blood loss, change in hemoglobin levels, hospitalization period, complications, postoperative white blood cell count, and postoperative C‐reactive protein (CPR) levels. Statistical analysis was performed using the Mann–Whitney *U* test and chi‐square test. The analysis performed in this study was by the Mann–Whitney *U* test because the sample size in this study does not allow for a normal distribution of the data, or the test is inadequate. All measured values were expressed as medians (range), with *p* values of < 0.1 indicating a trend, and *p* values of < 0.05 indicating a significant difference. This study was conducted with the approval of the Medical Research Ethics Committee of our university (HM23‐132).

## 3 Results

Patient characteristics of the SP‐RH group vs. SP + 1‐LH group were as follows, respectively: age (years) was 47 (36–55) versus 46 (36–54) (*p* = 0.562), body mass index (kg/m^2^) was 22.9 (18.8–32.5) versus 21.3 (15.1–31.8) (*p* = 0.079), parity (*n*) was 2 (0–3) versus 1 (0–3) (*p* = 0.013), number of previous cesarean sections (*n*) was 5 (25%) versus 7 (18%) (*p* = 0.594), and preoperative hemoglobin (g/dL) was 12.6 (7.8–15.4) vs. 13.3 (9.4–15.6) (*p* = 0.143). Except for the parity (*n*) (2 [0–3] vs. 1 [0–3] in SP‐RH group vs. SP + 1‐LH group, respectively), which was lower in the SP + 1‐LH group, no other significant differences were observed (Table 1).

**TABLE 1:** Demographic and clinical characteristics of the patients.

	SP‐RH (*n* = 20)	SP + 1‐LH (*n* = 37)	*p*
Age (years)	47 (36–55)^a^	46 (36–54)	0.562
Body mass index (kg/m^2^)	22.9 (18.8–32.5)	21.3 (15.1–31.8)	0.079
Parity (*n*)	2 (0–3)	1 (0–3)	**0.013**
Cesarean section	5 (25%)	7 (18%)	0.594
Preoperative hemoglobin (g/dL)	12.6 (7.8–15.4)	13.3 (9.4–15.6)	0.143

*Note:* Bold indicates statistically significant values, *p* < 0.05.

^a^ Data are the median (range) or number (%).

**FIGURE 3:**
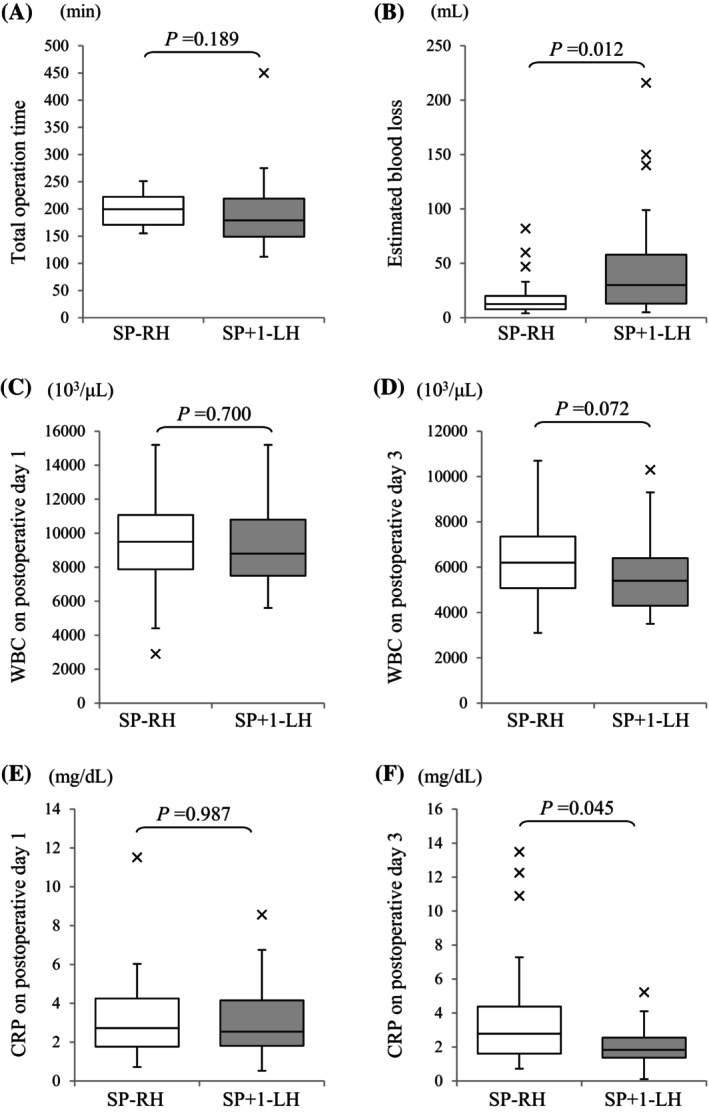
Comparative analysis of various surgical outcomes between the SP‐RH group and the SP + 1‐LH group. (A) Total operation time. (B) Estimated blood loss. (C) WBC count on Postoperative Day 1. (D) WBC count on Postoperative Day 3. (E) CRP level on Postoperative Day 1. (F) CRP level on Postoperative Day 3. The boxes indicate the 25th and 75th percentiles, whereas the bands near the middle indicate the median values. The bars indicate 1.5 interquartile ranges for specifically marked outliers. CRP, C‐reactive protein; WBC, White blood cell.

Furthermore, the surgery results were compared between the SP‐RH group and the SP + 1‐LH group: the weight of the specimen (g) was 218 (90–505) in the SP‐RH group versus 250 in the SP + 1‐LH group (50–655) (*p* = 0.134), the total operation time (min) was 200 in the SP‐RH group (155–251) versus 179 in the SP + 1‐LH group (112–450) (*p* = 0.189), the port placement time (min) was 8 in the SP‐RH group (3–19) versus 7 in the SP + 1‐LH group (3–13) (*p* = 0.607), the uterus out time (min) was 5 in the SP‐RH group (1–24) versus 11 in the SP + 1‐LH group (1–39) (*p* = 0.202), the estimated blood loss (mL) was 12 in the SP‐RH group (4–82) versus 36 in the SP + 1‐LH group (5–216) (*p* = 0.012), and the change of hemoglobin level (g/dL) was 0.1 in the SP‐RH group (−4.1 to 4.7) versus 0.7 in the SP + 1‐LH group (−0.7 to 2.7) (*p* = 0.062), demonstrating a greater reduction in hemoglobin level in the SP + 1‐LH group and a significant reduction in intraoperative blood loss in the SP‐RH group. Blood loss of more than 50 mL was observed in 10.0% and 24.3% in the SP‐RH group and SP + 1‐LH group (*p* = 0.132), respectively, and that of more than 100 mL was 0.0% and 8.1% in the SP‐RH group and SP + 1‐LH group (*p* = 0.191), respectively. Additionally, no differences were noted between the two groups in the incidence of intraoperative complications (0.0% vs. 2.7%) (*p* = 0.268) and the incidence of postoperative complications (40.0% vs. 16.2%) (*p* = 0.261). One intraoperative complication observed was bleeding due to omentum injury in the SP + 1‐LH group. Postoperative complications included five cases of bleeding at the stump requiring hospital visits and three cases of postoperative infection requiring antibiotic treatment among the SP‐RH group, and one case had postoperative ileus requiring conservative treatment, three cases of bleeding at the stump requiring hospital visits, and two cases of postoperative infection requiring antibiotic treatment among the SP + 1‐LH group. Moreover, no complications of Clavien–Dindo grade ≥ 3 occurred in either group. When postoperative inflammatory responses were compared, the white blood cell count (10^3^/μL) on the first postoperative day was 9.5 (2.9–15.2) versus 8.8 (5.6–15.2) (*p* = 0.700), the white blood cell count (10^3^/μL) on the third postoperative day was 6.2 (3.1–10.7) versus 5.4 (3.5–10.3) (*p* = 0.072), the CRP value (mg/dL) on the first postoperative day was 2.7 (0.7–11.5) versus 2.5 (0.5–8.6) (*p* = 0.987), and the CRP value (mg/dL) on the third postoperative day was 2.8 (0.7–13.5) versus 1.8 (0.1–5.2) (*p* = 0.045) in the SP‐RH group and SP + 1‐LH group, respectively, indicating that the CRP levels on the third postoperative day significantly decreased in the SP + 1‐LH group (Figure 3). Although significant differences were noted in the estimated blood loss and CRP value (mg/dL) on the third postoperative day, no significant differences in short‐term clinical outcomes were observed because the hospitalization period (days) was similar in both the SP‐RH group 7 (6–15) and the SP + 1‐LH group 7 (5–20) (*p* = 0.191) (Table 2).

**TABLE 2:** Surgical outcomes.

	SP‐RH (*n* = 20)	SP + 1‐LH (*n* = 37)	*p*
Weight of specimen (g)	218 (90–505)^a^	250 (50–655)	0.134
Total operation time (min)	200 (155–251)	179 (112–450)	0.189
Port placement time (min)	8 (3–19)	7 (3–13)	0.607
Uterus out time (min)	5 (1–24)	11 (1–39)	0.202
Estimated blood loss (mL)	12 (4–82)	36 (5–216)	**0.012**
Change of Hemoglobin (g/dL)	0.1 (−4.1 to 4.7)	0.7 (−0.7–2.7)	0.062
Hospitalization period (days)	7 (6–15)	7 (5–20)	0.191
Complications (*n*)	8 (40%)	7 (19%)	0.085
Intraoperative	0 (0.0%)	1 (2.7%)	0.268
Postoperative	8 (40.0%)	6 (16.2%)	0.261
Clavien–Dindo grade ≥ 3	0 (0%)	0 (0%)	
White blood cell count (10^3^/μL)			
Postoperative Day 1	9.5 (2.9–15.2)	8.8 (5.6–15.2)	0.700
Postoperative Day 3	6.2 (3.1–10.7)	5.4 (3.5–10.3)	0.072
C‐reactive protein (mg/dL)			
Postoperative Day 1	2.7 (0.7–11.5)	2.5 (0.5–8.6)	0.987
Postoperative Day 3	2.8 (0.7–13.5)	1.8 (0.1–5.2)	**0.045**

*Note:* Bold indicates statistically significant values, *p* < 0.05.

^a^ Data are the median (range) or number (%).

## 4 Discussion

The new da Vinci SP surgical system, which enables single‐port surgery, has advantages unique to robotic surgery, such as stable 3D images and precise surgical operations without the camera shaking. It also features a joint function that prevents interference between forceps, an issue inherent to single‐port laparoscopic surgery, allowing for good operability even in confined working spaces. In this study comparing single‐port plus one‐port laparoscopic surgery with single‐port robotic hysterectomy using the da Vinci SP, we observed a significant reduction in intraoperative blood loss in the SP‐RH group. Although the difficulty in surgical procedures for uterine tumors differs depending on the site and size of the tumor, the 3D magnified field of view characteristic of robotic surgery, stable surgical operation, and improved operability of the da Vinci SP may have contributed to the reduction in blood loss. However, no differences were observed in other surgical outcomes or complication rates. Regarding the postoperative inflammatory reaction, the CRP values on the third day after surgery were lower in the SP + 1‐LH group, suggesting that laparoscopic surgery may be less stressful on the wound. In the present study, postoperative infections in the SP‐RH group included cases with CRP as high as 13.5 mg/dL, which may have led to a significant difference. Also, the increased wound burden caused by the arm in robotic surgery could be an issue compared to laparoscopic surgery, even though the length of the abdominal incision is similar. There have also been reports of single‐port robotic surgery using the conventional 4‐arm da Vinci surgical system. One meta‐analysis observed that this modality significantly reduced blood loss compared to single‐port laparoscopic surgery [17]. Conversely, many reports have documented no significant differences in surgical outcomes or postoperative complications between the two modalities [18, 19, 20]. Gardella et al. [19] reported that there were no significant differences in surgical results between the two groups, but operating time and hospital stay were longer in the single‐port robotic surgery group than in the laparoscopic surgery group, and robotic surgery was associated with a higher risk of intraoperative bleeding. Also, Noh et al. [20] stated that the time required between the completion of colpotomy and the initiation of the vaginal stump suture is associated with the risk of increased bleeding. However, both cases were the result of single‐port surgery using the da Vinci Xi system; therefore, it could be attributable to settings and complexity or inexperience with procedures. Since the da Vinci SP is a platform developed specifically for single‐port robotic surgery, it is presumed that the da Vinci SP will improve operability and surgical outcomes compared to single‐port robotic surgery using the conventional 4‐arm da Vinci surgical system. Therefore, with the accumulation of future cases, the amount of blood loss, surgical time, and postoperative recovery may be improved by robotic surgery.

Moreover, when the outcomes were compared between single‐port robotic hysterectomy using the da Vinci SP and conventional multiport robotic hysterectomy using the da Vinci Xi at our institution, outcomes such as operation time, amount of blood loss, and postoperative complications were comparable [21]. Recently, Park et al. demonstrated that hysterectomy using the da Vinci SP may reduce blood loss [22]. Moreover, Matsuura et al. [23] have reported that it shortens operation time in early‐stage endometrial cancer. Therefore, with the advent of the da Vinci SP, the range of the application of the da Vinci SP is expected to expand from conventional multiport robotic surgery to single‐port robotic surgery.

Single‐port laparoscopic surgery involves complicated surgical procedures and requires time to master the technique, but the introduction of single‐port plus one‐port laparoscopic surgery has addressed these issues. The advantage of single‐port surgery is the cosmetic appearance of the wound; however, no clear conclusions have been reached regarding its invasiveness and patient satisfaction compared to laparoscopic surgery. The limitations of the present study are that it was performed using a small number of cases at a single center, and the long‐term prognosis and patient satisfaction are unknown. It should be noted that the potential bias may arise due to the retrospective study and the small number of participants in the SP‐RH group. Therefore, an appropriate study design, including patient background and sample size, as well as large‐scale validation by prospective design or randomized controlled trial (RCT), will be needed.

This study confirmed the safe implementation and feasibility of single‐port robotic hysterectomy using the da Vinci SP for benign gynecological and uterine diseases. A single‐port system seems particularly advantageous for patients in terms of esthetic outcomes and invasiveness. The da Vinci SP, a dedicated robotic surgical system, will especially help surgeons master the technique and improve clinical outcomes.

## Author Contributions

A.O. and H.N. drafted the article. A.O., K.T., A.K., M.I., R. I., H.M., and H.N. performed surgery as well as acquired and analyzed the data. All authors are in agreement with the content of the manuscript. All authors read and approved the final manuscript.

## Ethics Statement

The Ethics Committee of Fujita Health University Approved the study protocol (HM23‐132).

## Consent

This study was conducted using routinely recorded health data from patients who visited Fujita Health University. Patients were informed about the purpose and methods of the study through a website and were given the opportunity to opt out of the study if they did not want their data to be used for research purposes.

## Conflicts of Interest

The authors declare no conflicts of interest.

## Data Availability

The data that support the findings of this study are available from the corresponding author upon reasonable request.
